# Growth in early life predicts bone strength in late adulthood: The Hertfordshire Cohort Study

**DOI:** 10.1016/j.bone.2007.05.007

**Published:** 2007-09

**Authors:** Helen Oliver, Karen A. Jameson, Avan Aihie Sayer, Cyrus Cooper, Elaine M. Dennison, the Hertfordshire Cohort Study Group

**Affiliations:** MRC Epidemiology Resource Centre, University of Southampton, Southampton General Hospital, Southampton SO16 6YD, UK

**Keywords:** Bone, Strength, Density, Programming, Epidemiology

## Abstract

Infant growth is a determinant of adult bone mass, and poor childhood growth is a risk factor for adult hip fracture. Peripheral quantitative computed tomography (pQCT) allows non-invasive assessment of bone strength. We utilised this technology to examine relationships between growth in early life and bone strength.

We studied 313 men and 318 women born in Hertfordshire between 1931 and 1939 who were still resident there in adult life, for whom detailed early life records were available. Lifestyle factors were evaluated by questionnaire, anthropometric measurements made, and peripheral QCT examination of the radius and tibia performed (Stratec 4500).

Birthweight and conditional weight at 1 year were strongly related to radial and tibial length in both sexes (*p* < 0.001) and to measures of bone strength [fracture load *X*, fracture load *Y*, polar strength strain index (SSI)] at both the radius and tibia. These relationships were robust to adjustment for age, body mass index (BMI), social class, cigarette and alcohol consumption, physical activity, dietary calcium intake, HRT use, and menopausal status in women. Among men, BMI was strongly positively associated with radial (*r* = 0.46, *p* = 0.001) and tibial (*r* = 0.24, *p* = 0.006) trabecular bone mineral density (BMD). Current smoking was associated with lower cortical (radius: *p* = 0.0002; tibia: *p* = 0.08) and trabecular BMD (radius: *p* = 0.08; tibia: *p* = 0.04) in males. Similar trends of BMD with these anthropometric and lifestyle variables were seen in women but they were non-significant. Current HRT use was associated with greater female cortical (radius: *p* = 0.0002; tibia: *p* = 0.001) and trabecular (radius: *p* = 0.008; tibia: *p* = 0.04) BMD. Current HRT use was also associated with greater radial strength (polar SSI: *p* = 0.006; fracture load *X*: *p* = 0.005; fracture load *Y*: *p* = 0.02) in women. Women who had sustained any fracture since the age of 45 years had lower radial total (*p* = 0.0001), cortical (*p* < 0.005) and trabecular (*p* = 0.0002) BMD, poorer forearm bone strength [polar SSI (*p* = 0.006), fracture load *X* and *Y* (*p* = 0.02)], and lower tibial total (*p* < 0.001), cortical (*p* = 0.008), and trabecular (*p* = 0.0001) BMD.

We have shown that growth in early life is associated with bone size and strength in a UK population aged 65–73 years. Lifestyle factors were associated with volumetric bone density in this population.

## Introduction

Osteoporotic fractures are associated with considerable morbidity, mortality, and cost; recent estimates suggest a total cost of £1.7 billion in the United Kingdom alone [1]. Fracture risk ultimately depends on two factors: the mechanical strength of bone (determined by both bone size and volumetric density) and the forces applied to it. Epidemiological evidence has shown that growth in infancy is a determinant of bone mass (assessed by dual energy x-ray absorptiometry) in the third, sixth, and seventh decades [2–7] and that a low rate of growth in childhood is a risk factor for hip fracture in adulthood; hence for those individuals whose rate of childhood height gain was in the lowest quartile for the Finnish cohort studied, the hazard ratio for hip fracture was 1.9 (95% CI 1.1–3.2), compared with those in the highest quartile [8]. Recent technological advances have allowed non-invasive assessment of bone strength by means of peripheral quantitative computed tomography (pQCT). We utilised this new technology to examine relationships between both growth in early life and bone strength of the radius and tibia in 620 men and women from the Hertfordshire Cohort Study.

## Methods

In the late 1990s, 3000 men and women aged 60–75 years were recruited to a study, which was designed to examine the relationship between growth in infancy and the subsequent risk of adult disease, including osteoporosis (the Hertfordshire Cohort Study). The selection procedure for these individuals was as follows: in brief, with the help of the National Health Service Central Registry at Southport, and Hertfordshire Family Health Service Association, we traced men and women who were born during 1931–39 in Hertfordshire and still lived there during the period 1998–2003 [7]. The birthweight and weight at 1 year of age of each individual had been recorded in a ledger by a team of midwives and health visitors who had attended each birth in Hertfordshire in the 1930s and visited the child's home at intervals during the first year of life. Of this group, 498 men and 468 women from East Hertfordshire completed a questionnaire, attended a clinic visit, and underwent bone density measurement. Individuals were selected for bone density measurement by geographical area only (East Hertfordshire only) although men and women taking medication known to alter bone metabolism (bisphosphonates, oral steroids, and testosterone therapy) were excluded from the study. Women taking hormone replacement therapy (HRT) were allowed to participate, and adjustments made in the analysis; this was a pragmatic decision due to the large numbers of women in our cohort taking this medication.

In 2004–5, a follow-up study was performed in East Hertfordshire. The General Practitioners of subjects who had previously participated were contacted to ask if we could approach their patients again. Of the original cohort of 498 men and 468 women, 8 had died, 53 had moved away, and we were unable to obtain GP permission to approach a further 4 subjects. Forty seven patients were no longer on GP lists. Hence 437 men and 438 women were contacted by letter; of these 322 men (65%) and 321 women (69%) agreed to attend a local clinic, held at Welwyn Garden City.

At this clinic visit, a detailed lifestyle questionnaire was again administered to obtain lifestyle information regarding socio-economic status, medical history, cigarette smoking, alcohol consumption, and dietary calcium intake. Specific information was collected regarding significant medical events (including osteoporotic fracture) since the last clinic visit, and this information validated against GP records. Height was measured to the nearest 0.1 cm using a Harpenden pocket stadiometer (Chasmors Ltd, London, UK) and weight to the nearest 0.1 kg on a SECA floor scale (Chasmors Ltd, London, UK). Body mass index (BMI) was calculated as weight divided by height^2^ (kg/m^2^). The radial length was measured from the distal end of the ulna styloid to the tip of the olecranon in mm; the tibial length was measured from the prominence of the medial malleolus to the tibial plate in mm.

Peripheral quantitative computed tomography (pQCT) was performed of the radius and tibia (non-dominant side) using a Stratec 4500 instrument. A scout view was performed on the forearm and lower leg to identify a baseline for the measurements. The cortical end plate of the radius was used as an anatomical landmark. The reference line was fixed in the lateral, horizontal part of the radial cortical end plate. The middle of the distal cortical end of the tibia was used as a reference line. Two slices were taken in the forearm scan (4% and 66%); four slices were taken for the lower leg scan (4%, 14%, 38%, 66%). Measurement precision error, expressed as a coefficient of variation, ranged from 0.88% (tibial total density, 4% slice) to 8.8% (total radial area, 66% slice), but was typically around 1–3%. These figures were obtained by 20 volunteers who were part of the study undergoing 2 scans on the same day, the limb repositioned in the machine between examinations. The threshold for bone was set at 280 mg/cm^3^. Using this machine, 55% of the outer bone area is concentrically separated and defined as cortical; the remaining 45% is defined as trabecular bone. We chose to adopt this algorithm as it allowed better comparability for our work with other studies; in addition, as we hope to perform follow-up scans on this group, it allowed consistency in our approach (to use the peelmode function of the instrument may lead to difficulties with identification of the threshold value). Bone strength was estimated with respect to torsion (polar strength strain index or SSI) or bending with respect to the *X* or *Y* axis (fracture load *X* and fracture load *Y*). Measurements were made at 2 sites in the radius (4% and 66% slice) and in the tibia (4% and 38% slice).

The East and North Hertfordshire Ethical Committees granted ethical approval for the study and all participants gave written informed consent.

### Statistical methods

Normality of variables was assessed and variables transformed as required. The Stata statistical software package was used for the analyses. Pearson correlations and ANOVAs followed by multiple linear regression models were used to explore the role of lifestyle and anthropometric factors in determining bone mass. An unconditional centile for growth in the first year of life was calculated, prior to the calculation of centiles for weight at 1 year conditional on birthweight according to the methods of Cole [9]. This allowed us to examine relationships between adult bone mass and conditional centile for growth in the first year of life, conditional on centile at birth, hence accounting for regression to the mean in weights from birth to 1 year of age.

## Results

The characteristics of the study population at baseline are displayed in Table 1. The mean age of the men and women studied was 69.2 and 69.5 years respectively. The mean BMI was 27.2 and 27.8 in men and women respectively. The mean daily dietary calcium intake was 1248 mg and 1134 mg in men and women respectively. Thirty eight percent of the men and 63 percent of the women had never smoked, whilst 8% of the men and 5% of the women were current smokers. Five percent of men and 25% women were non-drinkers, whilst 21% of men and 11% of women were moderate drinkers (i.e. 11–21 units per week for men, 8–14 units per week for women). Fifty eight percent women in this cohort had never used hormone replacement therapy (HRT); 17% were currently taking HRT at the time of the study. The mean birthweight in this study was 3.05 kg and 3.04 kg in men and women respectively.

Among men, BMI was strongly positively associated with radial (*r* = 0.46, *p* = 0.001) and tibial (*r* = 0.24, *p* = 0.006) trabecular BMD. Current smoking was associated with lower cortical (radius: *p* = 0.0002; tibia: *p* = 0.08) and trabecular BMD (radius: *p* = 0.08; tibia: *p* = 0.04) in males. Similar trends of BMD with these anthropometric and lifestyle variables were seen in women but they were non-significant. Relationships between all pQCT measures and alcohol and dietary calcium consumption were weak in this population. Current HRT use was associated with greater female cortical (radius: *p* = 0.0002; tibia: *p* = 0.001) and trabecular BMD (radius: *p* = 0.008; tibia: *p* = 0.04). Current HRT use was also associated with greater radial strength (polar SSI: *p* = 0.006; fracture load *X*: *p* = 0.005; fracture load *Y*: *p* = 0.02) in women.

Table 2 shows the relationships between adult height and weight and pQCT measures. As anticipated, adult height was particularly associated with measures of bone length, whilst adult weight showed relationships with bone length and strength. Tables 3 and 4 show the correlation coefficients between early life parameters (birthweight and weight at 1 year) and pQCT measurements in this population. Radial and tibial length were strongly related to birthweight and conditional weight at 1 year in both sexes (*p* < 0.001); these relationships were robust to adjustment for adult weight and lifestyle but not adult height. Both birthweight and conditional weight at 1 year were related to measures of bone strength (fracture load *X*, fracture load *Y*, polar strength strain index) at both the radius and tibia. These relationships were robust to adjustment for age, social class, cigarette and alcohol consumption, physical activity, dietary calcium intake, HRT use, and menopausal status in women but were largely attenuated by adjustment for adult height and weight. Relationships were observed between radial cortical bone area and birthweight (robust to all adjustments) in men only. There were no relationships between early life and volumetric BMD in either sex. We also examined relationships between radial and tibial periosteal circumference, endosteal circumference, and cortical thickness with birthweight and conditional weight at 1 year. We observed relationships between tibial periosteal circumference and early life in both sexes that were robust to adjustment for adult height, weight, and lifestyle (birthweight: men *r* = 0.14, *p* < 0.05 women *r* = 0.19 *p* < 0.001; conditional weight at 1 year: men *r* = 0.18 *p* < 0.01 women *r* = 0.15 *p* < 0.05). While some relationships were also seen with endosteal circumference and early life, these were weaker and generally became non-significant after adjustments.

In this cohort, since the age of 45 years, 258 men had sustained no fractures, 16 men had sustained a fracture associated with major trauma (defined as a fall from greater than standing height) and 26 men had sustained a fracture associated with minor trauma (defined as a fall from standing height or less); corresponding figures amongst women were 262, 4, and 46 individuals. Women who had sustained any fracture since the age of 45 years had lower radial total (*p* = 0.0001), cortical (*p* < 0.005), and trabecular (*p* = 0.0002) BMD, in addition to poorer measures of forearm bone strength [polar SSI (*p* = 0.006), fracture load *X* and *Y* (*p* = 0.02)]. Similarly, they also had lower tibial total (*p* < 0.001), cortical (*p* = 0.008), and trabecular (*p* = 0.0001) BMD. Relationships were similar but non-significant in men.

## Discussion

We have shown that enhanced growth in early life is associated with greater bone size and strength as assessed by pQCT in a UK population in late middle age. Our results suggest that there is a small residual effect of early life, whereby poor growth produces weaker bone in adult life, even when adult height and weight are adjusted for. Birthweight and weight at 1 year displayed independent associations with measures of bone density and strength. Lifestyle factors such as cigarette consumption, HRT use, and obesity were associated with volumetric bone density in this population but did not explain the relationship between growth in early life and bone strength in late adulthood.

This study had a number of limitations. The individuals recruited were selected because they had been born in Hertfordshire and continued to live there at the age of 60–75 years, as in previous studies. However, we have previously demonstrated that the Hertfordshire populations studied have similar smoking characteristics and bone density to national figures [10], suggesting that selection bias is minimal. Furthermore, we ascertained that those individuals who did not complete the study had similar birthweights and weights at 1 year to those who did not. The power of the study to detect associations with fracture in men was low, due to low numbers of events.

Cadaveric studies have confirmed the high precision of pQCT and validated its use as a technique for assessing bone strength [11–14]. Cross-sectional studies of large cohorts with a wide age range have reported that total, trabecular, and cortical bone density decrease linearly with age in both sexes, with greater declines in females [15]. In women, cortical bone area decreases with age after the age of 60 years. The total cross-sectional area of the bone becomes wider with age, with greater increments in men than women, whilst the minimum moment of inertia, an index of mechanical resistance to bending, remains stable with age in men, whilst it was significantly lower in older compared with younger women. Case control studies have suggested that volumetric bone mineral content (BMC), total and trabecular volumetric BMD, and the cross-sectional moment of inertia are all significantly different in women who had sustained a low trauma Colles' fracture versus controls [16]. Similarly, one Japanese study has suggested that pQCT can be used to discriminate women who have sustained vertebral fractures versus non-fractured controls [17].

Initial studies from Bath showed a relationship between weight at 1 year and BMC at age 21 years in women [2], whilst further work in Hertfordshire, UK went on to demonstrate an association between weight at 1 year and BMC at age 60–75 years in both sexes [3]. More recent data from the US showed an association between recalled birthweight and BMC in 305 postmenopausal women [4]. Typically adjustment for adult weight or height weakens but does not remove this association. Similarly, data from Sheffield demonstrated significant associations between birthweight and adult BMC (and lean mass) after adjustment for age, sex, height, and physical activity [5]. Most recently, we have demonstrated independent contributions of birthweight and weight at 1 year to adult BMC and BMD in this cohort [7]. Available data would therefore support the hypothesis that different mechanisms exist for establishing the adult bone envelope which encompasses not only the length of the bone, but also its width (estimated by BMC) versus its density (estimated by BMD). Each of these factors is important in establishing an individual's fracture risk. The independent effects of birthweight and weight at 1 year suggest that although genetic and/or intrauterine environmental factors that influence the foetal growth trajectory and are reflected in birthweight have long-term consequences on bone mass in late adulthood, further modification of the infant growth trajectory over the first year of life has lasting effects on adult bone mass and hence fracture risk.

It is difficult to disentangle the influences of the genome and intrauterine environment on birthweight. In a family study performed 5 decades ago, Penrose suggested that 62% of the variation in birthweight between individuals was the result of the intrauterine environment, 20% was the result of maternal genes, and 18% was the result of foetal genes [18]. In addition, studies of babies born after ovum donation showed that although their birthweights were strongly related to the birthweights of the recipient mother, they were unrelated to the weight of the female donors [19]. Coupled with other studies [20–22], these data would suggest that birth size is controlled at least in part by the intrauterine environment rather than by the genetic inheritance from both parents. Finally, a recent study that examined the association of birthweight with bone mass in a twin study of over 4000 women confirmed that bone mass and especially BMC were highly associated with birthweight in both monozygotic and dizygotic twins [6]. These associations point to environmental rather than genetic factors underlying the observed relationships.

Adult bone mass is a function of both bone size and density [23]; both these variables influence fracture risk [24,25]. Both bone geometry and bone properties (including micro-architecture) are also known to be determinants of bone strength; with age, changes in the elastic modulus and toughness of bone are offset by periosteal apposition which may help to preserve bone strength [26]. BMD is limited at detecting fracture risk; although risk of fracture increases with decreasing BMD, many individuals fracture with a ‘normal’ BMD. In part, this may reflect the limitations of DXA in assessing bone density (by dividing BMC by projected bone area); projected bone area will systematically underestimate the skeletal bone volume of taller subjects. Hence pQCT provides a valuable additional tool.

In conclusion, we have demonstrated independent effects of birthweight and weight at 1 year on volumetric bone size and bone strength in the seventh decade in both sexes. Lifestyle factors such as cigarette smoking were the major determinants of volumetric bone density in this population.

## Figures and Tables

**Table 1:** Summary characteristics of study participants

Characteristic	Males (*n* = 313)	Females (*n* = 318)
*Mean (SD) unless stated otherwise*		
Age (years)	69.2 (2.5)	69.5 (2.6)
BMI (kg/m^2^)	27.2 (3.7)	27.8 (4.9)
Alcohol consumption (units per week)^a^	9 (2.3, 16.6)	3.0 (0.5, 7.0)
Habitual activity (%)^b^	64.0 (14.2)	61.8 (14.3)
Dietary calcium intake (mg/day)	1247.8 (320.7)	1134.4 (334.8)
Current manual social class (IIIM–V) *n* (%)^c^	169 (54.0)	182 (57.2)
Current non-manual social class (I–IIIN) *n* (%)^c^	128 (40.9)	136 (42.8)
Birthweight (kg)	3.05 (0.5)	3.04 (0.5)
Weight at 1 year (kg)	10.2 (1.1)	9.8 (1.1)

a Median and IQR amongst drinkers. 17 men and 78 women stated that they do not drink alcohol.

b Standardised score ranging 0–100 derived from frequency of gardening, housework, climbing stairs, and carrying loads in a typical week. Higher scores indicate greater level of activity.

c Social class was unclassified for 16 men. I–IIIN and IIIM–V denote classes one to three (non-manual), and three (manual) to five, of the 1990 OPCS Standard Occupational Classification scheme for occupation and social class. Social class was identified on the basis of own current or most recent full-time occupation for men and never-married women, but on the basis of the husband’s occupation for ever-married women.

**Table 2:** Relationships between pQCT measures and adult anthropometric measures

	Men		Women	
	Height	Weight	Height	Weight
Radius length	0.65***	0.31**	0.67***	0.39***
Radius total area	0.08	0.21**	0.19***	0.34***
Radius trabecular density	− 0.07	0.01	− 0.04	0.19
Radius cortical density	0.03	− 0.09	0.16***	0.09
Radius cortical bone area	0.14*	0.28***	0.32***	0.37***
Radius fracture load (*X*)	0.13*	0.26***	0.27***	0.25***
Radius fracture load (*Y*)	0.28***	0.26***	0.36***	0.28***
Radius strength strain index	0.21***	0.23***	0.34***	0.21***
Tibial length	0.76***	0.30***	0.75***	0.37***
Tibial total area	0.48***	0.36***	0.42***	0.41***
Tibial trabecular density	− 0.15*	0.14*	0.05	0.27
Tibial cortical density	0.03	− 0.12*	0.01	0.05
Tibial cortical bone area	0.34***	0.33***	0.39***	0.42***
Tibial fracture load *X*	0.37***	0.35***	0.46***	0.41***
Tibial fracture load *Y*	0.29***	0.41***	0.40***	0.48***
Tibial strength strain index	0.37***	0.38***	0.48***	0.45***

Figures are correlation coefficients. *p < 0.05; **p < 0.01; ***p < 0.001.

**Table 3:** Relationships between early life and radial pQCT measurements

	Men				Women			
	Birthweight (unadjusted)	Birthweight (adjusted)	Cond wt at 1 year (unadjusted)	Cond wt at 1 year (adjusted)	Birthweight (unadjusted)	Birthweight (adjusted)	Cond wt at 1 year (unadjusted)	Cond wt at 1 year (adjusted)
Radius length	0.25***	0.24***	0.24***	0.24***	0.25***	0.24***	0.24***	0.24***
Radius total area	0.14*	0.13*	0.14*	0.16**	0.19***	0.18**	0.07	0.08
Radius trab density	− 0.07	− 0.07	0.11	0.07	− 0.10	− 0.15*	− 0.02	− 0.06
Radius cort density	0.00	0.05	0.01	− 0.02	− 0.02	− 0.04	0.05	0.03
Radius cortical bone area	0.20***	0.12*	0.07	0.02	0.08	− 0.04	0.09	0.00
Radius fracture load (*X*)	0.14*	0.12*	0.06	0.08	0.08	0.06	0.11*	0.12*
Radius fracture load (*Y*)	0.14*	0.12*	0.12*	0.12*	0.09	0.08	0.12*	0.11*
Radial strength strain index	0.13*	0.10	0.11*	0.12*	0.08	0.07	0.12*	0.13

Figures given are Pearson correlation coefficients. *p < 0.05; **p < 0.01; ***p < 0.001. Adjustments made are age, BMI, cigarette and alcohol consumption, physical activity, dietary calcium intake, social class, HRT use, and years since menopause in women.

**Table 4:** Relationships between early life and tibial pQCT measurements

	Men				Women			
	Birthweight (unadjusted)	Birthweight (adjusted)	Cond wt at 1 year (unadjusted)	Cond wt at 1 year (adjusted)	Birthweight (unadjusted)	Birthweight (adjusted)	Cond wt at 1 year (unadjusted)	Cond wt at 1 year (adjusted)
Tibial length	0.17**	0.21***	0.27***	0.25***	0.23***	0.24***	0.24***	0.26***
Tibial total area	0.12*	0.13*	0.23***	0.23***	0.30***	0.29***	0.10	0.12
Tibial trab density	− 0.11	− 0.13*	0.07	0.03	− 0.05	− 0.10	0.05	0.01
Tibial cort density	0.02	0.04	− 0.02	− 0.03	− 0.03	− 0.05	0.03	0.02
Tibial cortical bone area	0.07	0.00	0.16**	0.01	0.19**	0.05	0.08	− 0.05
Tibial fracture load (*X*)	0.19**	0.18**	0.12*	0.09	0.16**	0.13*	0.16**	0.17**
Tibial fracture load (*Y*)	0.18**	0.15*	0.12*	0.10	0.18**	0.14*	0.16**	0.16**
Tibial strength strain index	0.19***	0.17**	0.12*	0.10	0.19**	0.15*	0.16**	0.17**

Figures given are Pearson correlation coefficients. *p < 0.05; **p < 0.01; ***p < 0.001. Adjustments made are age, BMI, cigarette and alcohol consumption, physical activity, dietary calcium intake, social class, HRT use, and years since menopause in women.
